# Phylogeography of a widely distributed plant species reveals cryptic genetic lineages with parallel phenotypic responses to warming and drought conditions

**DOI:** 10.1002/ece3.8103

**Published:** 2021-09-09

**Authors:** Sandra M. Kahl, Christian Kappel, Jasmin Joshi, Michael Lenhard

**Affiliations:** ^1^ Biodiversity Research/Systematic Botany Institute of Biochemistry and Biology University of Potsdam Potsdam Germany; ^2^ Berlin‐Brandenburg Institute of Advanced Biodiversity Research (BBIB) Berlin Germany; ^3^ Genetics Institute of Biochemistry and Biology University of Potsdam Potsdam Germany; ^4^ Institute for Landscape and Open Space Eastern Switzerland University of Applied Sciences Rapperswil Switzerland

**Keywords:** climate adaptation, ddRAD, *Silene vulgaris*

## Abstract

To predict how widely distributed species will perform under future climate change, it is crucial to understand and reveal their underlying phylogenetics. However, detailed information about plant adaptation and its genetic basis and history remains scarce and especially widely distributed species receive little attention despite their putatively high adaptability.

To examine the adaptation potential of a widely distributed species, we sampled the model plant *Silene vulgaris* across Europe. In a greenhouse experiment, we exposed the offspring of these populations to a climate change scenario for central Europe and revealed the population structure through whole‐genome sequencing. Plants were grown under two temperatures (18°C and 21°C) and three precipitation regimes (65, 75, and 90 mm) to measure their response in biomass and fecundity‐related traits. To reveal the population genetic structure, ddRAD sequencing was employed for a whole‐genome approach. We found three major genetic clusters in *S. vulgaris* from Europe: one cluster comprising Southern European populations, one cluster of Western European populations, and another cluster containing central European populations. Population genetic diversity decreased with increasing latitude, and a Mantel test revealed significant correlations between *F*_ST_ and geographic distances as well as between genetic and environmental distances. Our trait analysis showed that the genetic clusters significantly differed in biomass‐related traits and in the days to flowering. However, half of the traits showed parallel response patterns to the experimental climate change scenario. Due to the differentiated but parallel response patterns, we assume that phenotypic plasticity plays an important role for the adaptation of the widely distributed species *S. vulgaris* and its intraspecific genetic lineages.

## Graphical abstract

Twenty‐five European populations of *Silene vulgaris* were sequenced and exposed to a possible climate change scenario. The results reveal three cryptic genetic lineages in Europe with significant differences in morphological traits and parallel response patterns under climate change conditions. This suggests a compensatory strategy through phenotypic plasticity in the genetic lineages of *S. vulgaris*.

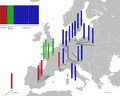

## 1 INTRODUCTION

Throughout their evolutionary history, organisms have had to cope with changing climates or other environmental changes. Species are able to deal with environmental changes through migration, phenotypic plasticity, and/or genetic adaptation (Exposito‐Alonso et al., 2018; Hämälä et al., 2018; Radchuk et al., 2019; de Villemereuil et al., 2018). However, as all species are limited by trade‐offs and only possess a certain range of tolerable environmental conditions, rapid climate change may represent an intensive threat that affects their survival (Barnosky et al., 2011; Pacifici et al., 2015; Radchuk et al., 2019; Trisos et al., 2020).

To reliably predict biodiversity changes under climate change, it is also important to take a closer look at the phylogeography of species as genetic lineages on an intraspecific level may differ in their adaptive genetic responses (Prunier et al., 2012; Schwarzer & Joshi, 2017). Past distribution patterns are often reflected in current species phylogenies and can be associated with divergent environmental conditions. This ghost of selection past (Samani & Bell, 2016) may be a strong selective force leading to differing genetic adaptations (García‐Fernández et al., 2013; Prunier et al., 2012). Especially widely distributed species consist of a variety of populations that can show morphological differences or exhibit local adaptation (Joshi et al., 2001; Pearman et al., 2010; Wright et al., 2006). So far, only few studies have dealt with the adaptation potential or response differences on an intraspecific level; as a result, little is known about the implications of ignoring phylogeographic structures when studying climate change responses (Pearman et al., 2010; Pfenninger et al., 2007). Studies on *Pinus* and other widely distributed species show that differences in intraspecific response to various climatic factors can be found and should be of importance when studying the impacts of climate change (Brabec et al., 2017; Oney et al., 2013; Rehfeldt et al., 2002; Zhang et al., 2004).

The herbaceous plant *Silene vulgaris* is such a widely distributed species that covers a south‐north gradient from North Africa up to the far North of Europe and a west‐east gradient from Iceland to the Middle East and temperate Asia; the species was also introduced to North America, Australia, South Africa, Ethiopia, and Japan (Registry‐Migration.Gbif.Org, 2021; WFO, 2021). *S. vulgaris* possesses a variety of known ecotypes especially adapted to extreme environmental conditions (i.e., heavy‐metal soil pollution) (Muszyńska et al., 2019; Pacwa‐Płociniczak et al., 2018). Furthermore, we reported in an earlier study that *S. vulgaris* responds considerably toward climatic changes through phenotypic plasticity (Kahl et al., 2019a). These characteristics make *S. vulgaris* a suitable species to investigate the response differences of genetic lineages to climate change. To examine these response differences, we sampled 25 European populations of *S. vulgaris* spanning a latitudinal gradient and tested the response of the different genetic lineages to a simulated climate change scenario for central Europe. Population structure was phylogeographically evaluated using ddRAD sequencing, and populations were exposed to a potential climate change scenario (with a temperature increase by 3°C and a reduced precipitation by 15 and 25 mm per summer month, respectively) to examine their phenotypic response. For the evaluation of phenotypic responses, nine different plant traits were measured. We chose those plant traits that are known to strongly react to temperature and precipitation changes and are proxies for plant fitness (Eziz et al., 2017; Hatfield & Prueger, 2015; Memmott et al., 2007; Wellstein et al., 2017).

It was the aim of the study (a) to reveal the genetic population structure of the sampled *S. vulgaris* populations across Europe and (b) to test whether putatively different genetic lineages of *S. vulgaris* showed a different response to a simulated climate change scenario for central Europe.

## 2 MATERIALS AND METHODS

### 2.1 Model plant species, sample collection, and greenhouse experiment

*Silene vulgaris* (Moench) Garcke is a diploid (2n = 24), perennial plant of the family of Caryophyllaceae with a self‐compatible reproductive system. Its native range covers the entire European continent including islands and expands toward Russia, North Africa (Morocco, Egypt), to the Middle East and parts of Asia (Bushneva, 2002; Pearl et al., 2009; Registry‐Migration.Gbif.Org, 2021; Taylor & Keller, 2007). Apart from its native ranges in Eurasia, *S. vulgaris* has also colonized North America where it has become an invasive species in some locations (McCauley et al., 2003; Taylor & Keller, 2007). Populations of *S. vulgaris* consist of female and hermaphrodite individuals making it a gynodioecious plant. It typically occurs in open grasslands or cultivated fields as well as on abandoned lots and can exhibit a high heavy‐metal tolerance (Bringezu et al., 1999; Taylor & Keller, 2007). The pollination of *S. vulgaris* is primarily done by moths, bumble bees, and hover flies (Jürgens et al., 1996; Pettersson, 1991). For the present study, a total of 325 plants were sampled in 2015 along a latitudinal gradient in Europe (Table 1). The plants belonged to 25 different populations of which seed samples were collected from 13 open pollinated plants per population. One of the populations (F3) was collected at three different locations at the southern coast of France (Figure 3). The sampling of each of the locations did not result in sufficient seed material for a balanced experimental design. Hence, we combined the seeds from the different locations into one single population (F3) as we considered the coastal area of France an important addition to the experiment based on the different climate conditions. Six seeds per plant were grown in a greenhouse and exposed to two different constant temperatures (18°C and 21°C) and three different precipitation conditions (90, 75, and 65 mm per month*)* as described *in* Kahl et al. (2019a). The temperatures were held constant using a setup of heating mats (“BioGreen WP 030‐060”; Bio Green OHG, Germany) and thermostats (“Universal UT 200‐2”; manufacturer: ELV Elektronik AG, Germany, Kahl et al., 2019a). For a precise watering, a commercial bottle‐top dispenser was used, and each pot was watered individually. The conditions resembled a possible climate change scenario for central Europe with increased average annual temperatures and decreased rainfall (Ahlström et al., 2012; IPCC, 2013). The following fitness‐related plant traits were measured to assess the performance under the experimental conditions: germination, survival, flowering, biomass, plant height, days to flowering, number of flowers, number of branches, number of leaves, leaf area, and specific leaf area (SLA). These plant trait data have been already used in an earlier analysis in Kahl et al. (2019b), but the current publication extends these findings by including a population genomic analysis via ddRAD sequencing.

**TABLE 1:** Summary of locations and genetic analyses of the *Silene vulgaris* populations studied (altitude in m a.s.l.)

Population ID	Site	Altitude [m]	Latitude	Longitude	*F*_IS_	*H*_e_	*H*_o_	*π*
A1	Gschnitz	1,234	47°02′51.0″N	11°21′29.0″E	0.002	0.039	0.058	0.060
CH1	Flims	2,102	46°52′03.8″N	09°14′23.4″E	0.026	0.051	0.069	0.084
CH2	Cadenazzo	202	46°09′09.0 ″N	08°56′33.0″E	0.014	0.054	0.071	0.079
D1	Berlin	30	52°31′58.7″N	13°23′10.2″E	0.053	0.069	0.067	0.096
D2	Hamburg	25	53°40′21.0″N	10°05′04.8″E	0.025	0.056	0.065	0.079
D3	Berlin	46	52°28′14.4″N	13°23′44.4″E	0.046	0.064	0.063	0.088
D5	Mainz	89	49°59′53.4″N	08°13′15.7″E	0.027	0.048	0.053	0.068
D6	Heilsbronn	400	49°20′44.6″N	10°47′26.5″E	0.004	0.038	0.056	0.059
D7	Gerswalde	70	53°11′06.3″N	13°45′28.0″E	0.031	0.054	0.059	0.076
D8	Geesower Hügel	30	53°14′32.9″N	14°23′06.9″E	0.037	0.048	0.047	0.068
D9	Potsdam	90	52°21′44.6″N	13°04′34.9″E	0.022	0.044	0.051	0.064
D10	Konstanz	400	47°40′23.0″N	09°09′10.6″E	0.002	0.043	0.068	0.069
D11	Hamm	90	49°44′26.2″N	08°26′58.6″E	0.029	0.060	0.070	0.087
D12	Langenargen	402	47°36′33.2″N	09°31′43.7″E	0.016	0.051	0.063	0.072
E1	Alcanó	211	41°29′29.4″N	00°26′27.3″E	−0.006	0.075	0.119	0.117
E2	Fontdepou	798	41°57′44.6″N	00°45′51.4″E	−0.009	0.064	0.107	0.102
E3	La Sentiu de Sió	316	41°49′44.1″N	00°54′19.9″E	−0.022	0.072	0.118	0.106
E4	La Palma	1,100	28°46′58.0″N	17°56′22.0″W	−0.009	0.067	0.103	0.099
F1	Les Rochette	65	46°41′00.8″N	01°23′58.0″W	0.011	0.042	0.057	0.064
F2	Pommeraie	78	43°56′42.6″N	01°22′21.5″E	0.027	0.052	0.055	0.070
F3	Menton	73	43°47′13.7″N	07°29′58.2″E	−0.008	0.059	0.092	0.087
	La Ciotat	44	43°11′55.2″N	05°37′53.3″E				
	Forcalquier	554	43°56′42.6″N	05°48′34.9″E				
F4	La Noue du Bourg	177	46°39′28.0″N	01°23′13.6″W	0.023	0.051	0.063	0.077
F5	Normandie	83	49°16′06.0″N	01°37′39.0″E	0.020	0.049	0.058	0.069
S1	Södra Bäck	10	56°40′12.7″N	16°40′54.2″E	0.044	0.066	0.069	0.094
S2	Vickleby	51	56°34′37.1″N	16°27′39.5″E	0.021	0.049	0.060	0.073

Note

*H*_o_: observed heterozygosity, *H*_e_: expected heterozygosity, *F*_IS_: inbreeding coefficient, *π*: Nucleotide diversity.

### 2.2 ddRAD library preparation and sequencing

For the extraction of genomic DNA, leaf tissue samples were taken from 13 randomly chosen individuals from each population in the greenhouse. Therefore, each mother plant was represented by six half‐sib progenies. The tissue samples were dried in silica gel until further processing. Genomic DNA was extracted from the samples using Qiagen DNeasy Plant Mini Kit (Qiagen, Hilden, Germany). The library preparation was performed as in Peterson et al. (2012) with some modifications. Double‐digest reactions were carried out in a volume of 100 μl containing ~500 ng of genomic DNA, 20 U of MspI and EcoRI, and 10× CutSmart^®^ buffer (NEB, Frankfurt am Main, Germany). Individual adapters were ligated on to 50 ng of digested DNA for a final pooling of 48 individuals (Table S1). Size selection was carried out using Pippin Prep targeting fragments between 276 and 476 bp. Multiplexing indices (6 bp) and Illumina sequencing primers were added to ~20 ng of size‐selected sample and CloneAmp™ HiFi PCR Premix (Takara, Saint‐Germain‐en‐Laye, France) in a final PCR amplification. Each sample was amplified in a 20 μl reaction volume with 14 cycles following the manufacturer's protocol. Sample libraries were pooled in equal amounts and quantified using Agilent 2200 TapeStation System (Agilent Technologies, Waldbronn, Germany). Paired‐end sequencing was performed using Illumina NextSeq 500 System (Illumina, München, Germany).

### 2.3 RAD‐seq data analysis and SNP identification

Raw Illumina reads were demultiplexed by their unique barcode and adapter sequences into unique reads for each individual using the *process_radtags* command in STACKS (v1.47) (Catchen et al., 2013). Reads were shortened to 140 bp to obtain equal length. ddRAD‐seq loci were assembled using the *de novo* pipeline ustacks, cstacks, sstacks, tsv2bam, and gstacks in STACKS (v2.4) (Rochette et al., 2019) due to the lack of a reference genome. Programs were run with the following parameters: ustacks ‐t gzfastq ‐f [sample].1.fq.gz ‐i [number] ‐‐name [sample] ‐o stacks/ ‐p 22 (for each sample separately); cstacks ‐P stacks/ ‐M population‐map.txt ‐n 4 ‐p 22, and sstacks ‐P stacks/ ‐M population‐map.txt ‐p 22; tsv2bam ‐P stacks/ ‐M population‐map.txt ‐‐pe‐reads‐dir fastq/ ‐t 22; gstacks ‐P stacks/ ‐M population‐map.txt ‐t 22. SNP calling took place at the gstacks step.

### 2.4 Population genetic analyses and genetic structure

The populations program in STACKS (*v.2.4*) was used to calculate observed heterozygosity (*H*_o_), expected heterozygosity *(H*_E_ *),* inbreeding coefficient of populations (*F*_IS_), nucleotide diversity (*π*), and the interpopulation component *F*_ST_. Population structure was estimated using fastSTRUCTURE (Raj et al., 2014) with simple prior. Best model complexity from 2 to 7 was chosen using its chooseK.py program.

We calculated a population‐level phylogenetic tree based on nucleotides fixed within populations and variant among them obtained using the ‐‐phylip parameter of the populations program in STACKS (v2.4). The phylogenetic analysis was done using the maximum likelihood approach in RAxML (v8.2.9) (Stamatakis, 2014). We employed a GTR + gamma model of sequence evolution for single full ML tree searches. Nodal support of the phylogenetic tree was evaluated by 500 replicates of RAxML's rapid bootstrap algorithm. The robustness of the obtained phylogenetic tree is indicated by the 100% bootstrap support for the three major branches.

### 2.5 Genetic and environmental differentiation

To examine possible genetic and environmental correlations among sampled populations, Mantel tests were performed. Climate variables of population sampling sites were acquired from the WorldClim database from 1970 to 2000 (Fick & Hijmans, 2017) and are listed in the appendix (Table S2). The climate data included annual mean temperature, mean diurnal temperature range, isothermality, temperature seasonality, maximum temperature in warmest month, minimum temperature in coldest month, temperature annual range, mean temperature of wettest, driest, warmest, and coldest quarter, annual mean precipitation, precipitation of wettest and driest month, precipitation seasonality, precipitation of wettest, driest, warmest, and coldest quarter, solar radiation, wind speed, and water vapor pressure. The data represent average values from the years 1970–2000. With this comprehensive approach, we aimed to account for the large distribution of samples across Europe and potential differences in climatic zones. R (version 3.4.3; R Core Team, 2017) was used to calculate dissimilarity matrices between population sampling sites of *S. vulgaris* based on the climate data listed above and pairwise geographic distances. Geographic distances between sampling sites were calculated with the R package geosphere and transformed into Euclidean distances. Environmental variables were normalized due to varying units and scales using the scale function in R, and Euclidean distances were calculated between populations. Mantel tests were performed between environmental and genetic distances (expressed as *F*_ST_), geographic and genetic distances, and environmental and geographic distances with 100,000 repetitions (Table 2).

**TABLE 2:** Genetic differentiation (*F*_ST_) of 25 *Silene vulgaris* populations

	A1	CH1	CH2	D1	D2	D3	D5	D6	D7	D8	D9	D10	D11	D12	E1	E2	E3	E4	F1	F2	F3	F4	F5	S1	S2
A1	–																								
CH1	0.32	–																							
CH2	0.26	0.25	–																						
D1	0.24	0.25	0.21	–																					
D2	0.28	0.29	0.24	0.22	–																				
D3	0.25	0.26	0.21	0.18	0.22	–																			
D5	0.31	0.30	0.23	0.21	0.26	0.22	–																		
D6	0.28	0.28	0.22	0.19	0.24	0.20	0.23	–																	
D7	0.26	0.27	0.22	0.18	0.23	0.19	0.21	0.20	–																
D8	0.29	0.30	0.23	0.21	0.25	0.22	0.24	0.23	0.22	–															
D9	0.29	0.30	0.23	0.19	0.25	0.21	0.25	0.22	0.21	0.24	–														
D10	0.29	0.29	0.22	0.19	0.24	0.20	0.24	0.22	0.20	0.23	0.23	–													
D11	0.25	0.25	0.21	0.19	0.22	0.20	0.20	0.20	0.20	0.21	0.21	0.20	–												
D12	0.27	0.28	0.22	0.20	0.24	0.21	0.24	0.22	0.21	0.24	0.23	0.22	0.20	–											
E1	0.29	0.29	0.25	0.25	0.26	0.25	0.26	0.25	0.26	0.27	0.27	0.26	0.23	0.26	–										
E2	0.31	0.29	0.25	0.24	0.26	0.23	0.27	0.24	0.25	0.27	0.27	0.27	0.22	0.26	0.18	–									
E3	0.32	0.31	0.27	0.26	0.28	0.27	0.28	0.27	0.28	0.29	0.29	0.29	0.23	0.28	0.19	0.22	–								
E4	0.31	0.31	0.26	0.26	0.27	0.27	0.28	0.26	0.27	0.28	0.29	0.27	0.24	0.28	0.19	0.22	0.22	–							
F1	0.33	0.28	0.27	0.26	0.30	0.27	0.31	0.29	0.29	0.31	0.30	0.31	0.26	0.29	0.29	0.29	0.31	0.31	–						
F2	0.32	0.27	0.28	0.27	0.30	0.27	0.31	0.29	0.29	0.32	0.30	0.31	0.27	0.29	0.29	0.29	0.31	0.31	0.21	–					
F3	0.29	0.28	0.24	0.25	0.24	0.25	0.26	0.24	0.26	0.27	0.27	0.26	0.23	0.26	0.20	0.21	0.22	0.22	0.28	0.29	–				
F4	0.32	0.24	0.27	0.26	0.28	0.27	0.30	0.29	0.28	0.30	0.30	0.30	0.26	0.29	0.26	0.27	0.29	0.29	0.22	0.22	0.24	–			
F5	0.30	0.27	0.26	0.24	0.27	0.25	0.28	0.27	0.27	0.29	0.28	0.28	0.24	0.27	0.27	0.27	0.29	0.29	0.25	0.24	0.26	0.25	–		
S1	0.24	0.26	0.21	0.19	0.21	0.19	0.23	0.20	0.19	0.21	0.21	0.20	0.20	0.21	0.25	0.24	0.27	0.27	0.27	0.28	0.25	0.27	0.26	–	
S2	0.26	0.27	0.22	0.18	0.22	0.19	0.23	0.21	0.19	0.22	0.21	0.20	0.20	0.21	0.25	0.25	0.28	0.27	0.28	0.28	0.25	0.28	0.26	0.17	–

### 2.6 Trait differentiation analysis toward climate change

The three main groups revealed by the population genetic analysis through STRUCTURE were included in the analysis of phenotypic changes related to a possible climate change scenario. Using different mixed models, we tested for phenotypic differences in *S*. *vulgaris* between our experimental climate change conditions and between the three genetic clusters revealed through the population genetic analysis (see above). Linear mixed models were employed for the statistical analyses of plant traits that fitted a normal distribution (biomass, plant height, days to flowering, leaf area, and specific leaf area). For binary data (plant survival and flowering) and count data (number of flowers, number of leaves, number of branches), we used generalized mixed models with a binomial and Poisson distribution, respectively. Furthermore, we also tested whether the genetic clusters responded differently to the climate change conditions by including the interaction term (Temp × Cluster; Prec × Cluster; Table 3) in our analysis. Models were performed using the function lmer() and glmer() in R (R Core Team, 2017). We included experimental treatment (divided in temperature and precipitation), cluster affiliation, temperature × precipitation, temperature × cluster affiliation, and precipitation × cluster affiliation as fixed factors (Table 3). Random factors were population identity and mother plant. Differences in germinating seed numbers were evaluated through Welch's two‐sample *t* test due to unequal sample sizes in the three genetic clusters. The influences of experimental conditions on germination could not be tested as treatments started two weeks after germination to ensure a maximum germination and seedling survival rate. The analysis via mixed‐effect models revealed significant effects of the precipitation and temperature treatments on *S. vulgaris* traits (Table 3). Details of this influence were not within the scope of the present study and have been discussed further in Kahl et al. (2019a). The aim of the present study is to analyze trait differences in relation to the phylogenetic relationship between *Silene* populations.

**TABLE 3:** ANOVA results of plant phenotypic traits in *Silene vulgaris* grown under two different temperature and three different precipitation conditions

	*df*	Height		Flower number		Leaf number		Branch number		Leaf area	
		*χ* ^2^	*p*	*χ* ^2^	*p*	*χ* ^2^	*p*	*χ* ^2^	*p*	*χ* ^2^	*p*
Temp	1	**46.3**	**<.001**	**539.0**	**<.001**	**56.3**	**<.001**	**9.5**	**<.01**	**15.1**	**<.001**
Prec	2	**43.8**	**<.001**	**147.5**	**<.001**	**131.8**	**<.001**	**18.5**	**<.001**	4.2	.12
Cluster	2	**26.5**	**<.001**	**11.6**	**<.01**	**8.3**	**<.05**	**20.9**	**<.001**	**9.1**	**<.05**
Temp × Prec	2	1.7	.42	**9.6**	**<.01**	**23.4**	**<.001**	1.2	.56	0.0	.98
Temp × Cluster	2	**7.8**	.**01**	4.0	.14	**9.9**	**<.01**	**7.5**	**<.05**	**6.3**	**<.05**
Prec × Cluster	4	8.7	.07	**11.8**	**<.05**	**33.3**	**<.001**	**11.1**	**<.05**	1.1	.89
*R* ^2^ marginal (conditional)		0.27 (0.46)		0.29 (0.85)		0.20 (0.63)		0.12 (0.21)		0.10 (0.33)	

	*df*	Germination		Survival		Flowering		Biomass		Days to flowering		SLA	
		*t*	*p*	*χ* ^2^	*p*	*χ* ^2^	*p*	*χ* ^2^	*p*	*χ* ^2^	*p*	*χ* ^2^	*p*
Temp	1	–	–	0.1	.75	**25.3**	**<.001**	**94.7**	**<.001**	**6.0**	.**01**	**6.4**	.**01**
Prec	2	–	–	1.3	.53	1.4	.50	**172.9**	**<.001**	0.3	.85	5.5	.06
Cluster	2	**13.0**	**<.01**	2.1	.34	2.4	.30	**4.7**	**<.05**	**7.1**	**<.05**	0.6	.73
Temp × Prec	2	–	–	1.5	.48	1.4	.50	0.4	.83	1.6	.46	0.0	.99
Temp × Cluster	2	–	–	0.6	.73	0.9	.64	0.2	.95	1.0	.60	1.9	.38
Prec × Cluster	4	–	–	2.6	.62	4.6	.33	0.4	.95	0.9	.92	2.1	.71
*R* ^2^ marginal (conditional)		0.11 (0.29)		0.01 (0.06)		0.04 (0.20)		0.14 (0.31)		0.07 (0.30)		0.01 (0.12)	

Note

Cluster refers to the genetic cluster affiliation revealed for the *S*. *vulgaris* populations in the phylogenetic analysis (Figure 2). Significant effects are indicated in bold. For binary data (germination, flowering, survival), a binomial distribution and for count data (number of flowers, leaves, branches), a Poisson error distribution was assumed.

Abbreviations: *df*, degree of freedom; SLA: Specific leaf area.

## 3 RESULTS

### 3.1 Summary statistics

**FIGURE 1:**
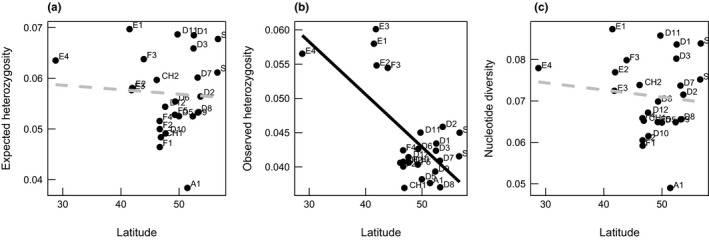
Relationship between (a) expected heterozygosity and latitude (*p* > .05), (b) observed heterozygosity and latitude (*p* < .001), (c) nucleotide diversity (*π*) and latitude (*p* > .05) of 25 European *Silene vulgaris* populations

Illumina sequencing of the ddRAD libraries provided 286 million fragments. Sample average and median were 971,650 and 767,040, respectively. The inbreeding coefficient (*F*_IS_) was close to zero for all populations ranging from 0.024 (A1) to 0.087 (D1; Table 1), hence not providing evidence for inbreeding. Expected heterozygosity ranged from 0.038 (A1) to 0.070 (E1; Table 1). Observed heterozygosity of populations ranged from 0.037 (CH1, D11) to 0.060 (E3) (Table 1). Values for nucleotide diversity of the different populations ranged from 0.049 (A1) to 0.087 (E1). The spatial distribution of population heterozygosity revealed a significant latitudinal pattern only for observed heterozygosity (Figure 1). Observed heterozygosity declined significantly with higher latitudes (Figure 1b; *p* < .001). We also found a trend toward a decline of expected heterozygosity and nucleotide diversity with increasing latitude; however, this was not significant (Figure 1a,c).

### 3.2 Population genetic structure and analysis

**FIGURE 2:**
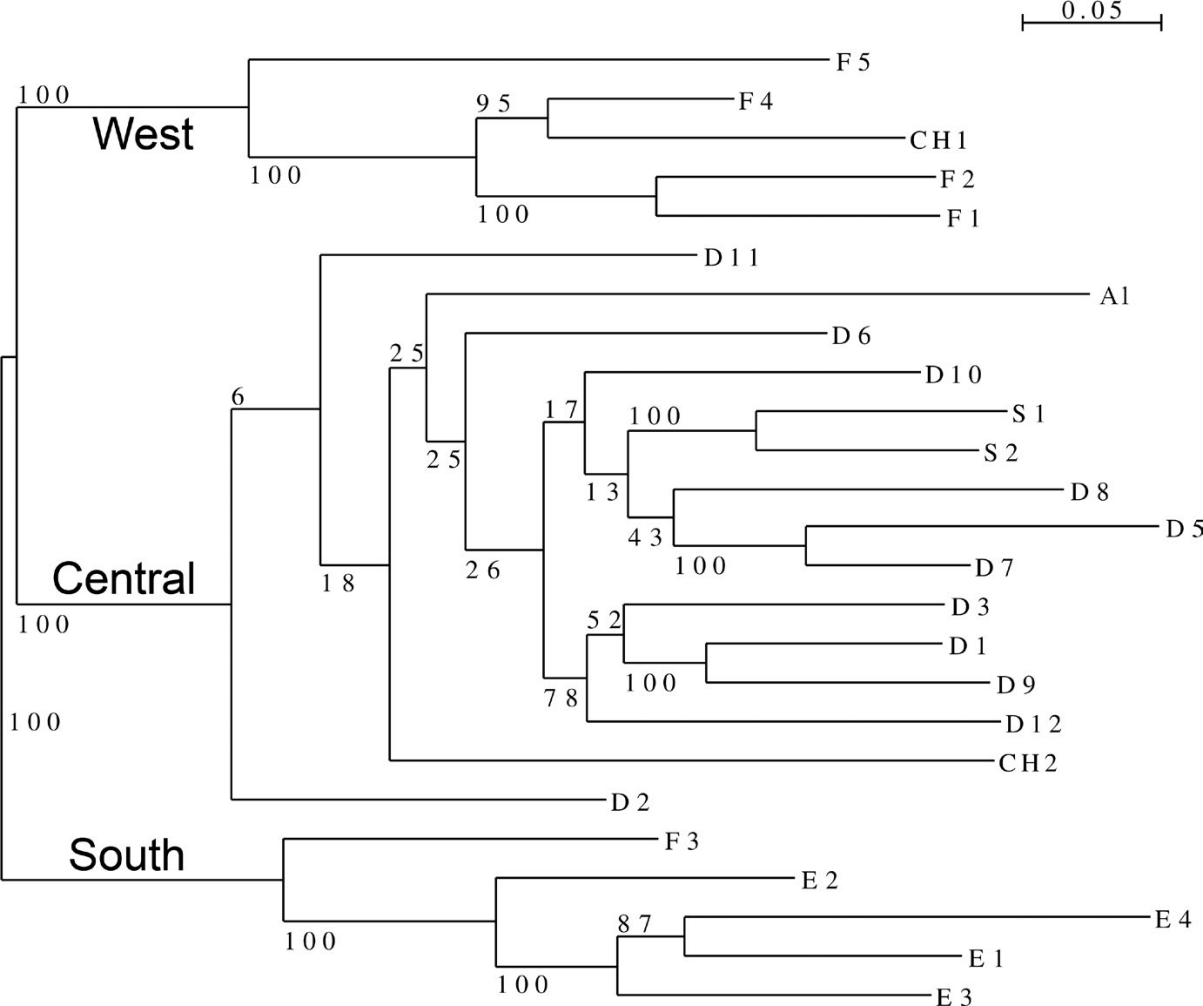
Maximum likelihood tree for the 25 *populations* of *S. vulgaris* generated by RAxML. Numbers represent bootstrap values (in %) from 500 replicates. Three major clusters were identified and used for further analysis of plant traits: “Central” = central‐north European cluster, “West” = West European cluster and “South” = South European cluster

The phylogenetic analysis revealed three major clusters among the European *S*. *vulgaris* populations sampled. All Spanish (E1, E2, E3, E4) and one southern French population (F3) formed one major monophyletic cluster of South‐Western Europe (hereafter referred to as cluster “South”), whereas the second monophyletic branch comprised the German (D1, D2, D3, D5, D6, D7, D8, D9, D10, D11, D12), Austrian (A1), Swedish (S1, S2), and one Southern Swiss population (CH2) of central Europe (hereafter referred to as cluster “Central”*)*. The third cluster will be referred to as cluster “West” comprising most French populations (F1, F2, F4, F5) and the remaining Swiss alpine population (CH1; Figure 2 *)*. Within the South cluster, the Spanish populations formed a monophyletic group with one French population as a sister group with 100% bootstrap support. The central European populations of *S. vulgaris* formed one distinct cluster with comparably low resolution. Within this cluster, no further geographic differentiation was possible with the data available, and the position of each population showed low bootstrap support. We found two Swedish populations (S1 and S2) that formed sister groups (S1 and S2) as well as many of the German populations (e.g., D1 and D9 from Potsdam and Berlin). However, the remaining genetic relationships did not correspond to a geographic pattern within this central European cluster (Figure 2). In the “West” cluster, the Swiss alpine population CH1 was nested among the French populations.

**FIGURE 3:**
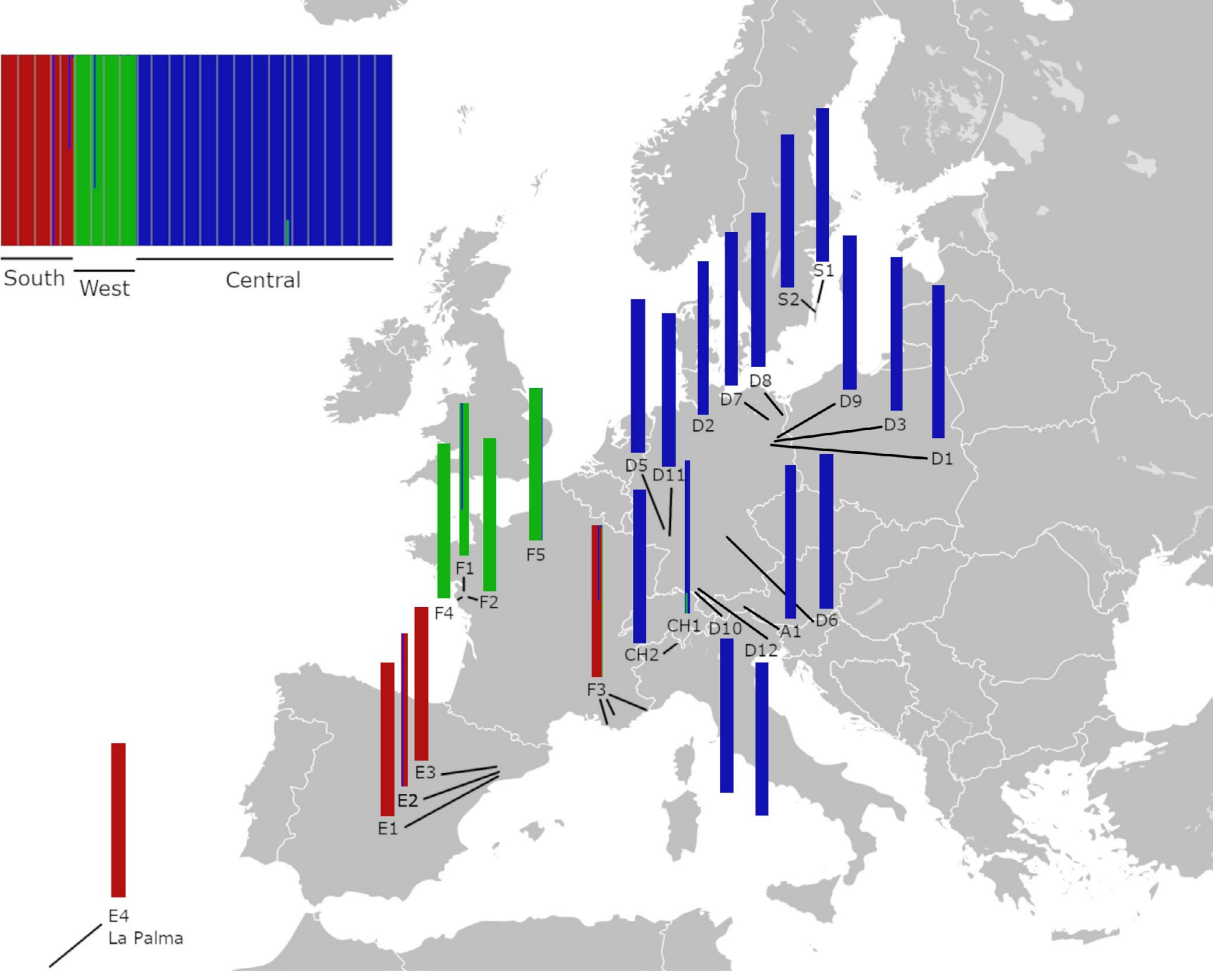
Multivariate analysis via ddRAD sequencing of 25 *Silene vulgaris populations*. The map shows the locations of the sampled populations including their population genetic structure. The different populations cluster in three major groups referred to as “South,” “Central,” and “West”

The multivariate analysis of SNPs using STRUCTURE largely agrees with the phylogenetic structure (Figure 3). At *K* = 3, cluster “South” is identical with populations E1, E2, E3, E4, and F3. The remaining clusters from the STRUCTURE analysis only differ slightly by including the Swiss alpine population CH1 in the “Central” cluster instead of the “West” cluster. As this clustering based on STRUCTURE more closely reflects the geographical proximity of populations, we used these three clusters for the further analyses below.

In our additional analysis through the pairwise *F_ST_* value comparison, a very similar pattern to the phylogenetic analysis was found (Figure S1): Here, the “South” cluster is identical comprising populations E1, E2, E3, E4, and F3 (Figure 2). However, it shows that populations CH1 and A1 both cluster in the “West” cluster together with the remaining French populations. Apart from these differences, the third cluster in the pairwise *F*_ST_ value comparison included the same populations as in the phylogenetic analysis (D1, D2, D3, D5, D6, D7, D8, D9, D10, D11, D12, and CH2; Figure S1). The three groups had a significantly higher genetic similarity within than among each other.

### 3.3 Mantel tests and population differentiation

The Mantel tests revealed a strong genetic pattern for the correlation between environmental, geographic, and genetic distances (Figure S2). Based on the climate data analyzed from the populations' sites (Table S2), we found a significant positive correlation of geographic and environmental distances with *r = *0.89 *and p* < .001 (Figure S2a). Furthermore, Mantel tests resulted in significant correlations between *F_ST_* and geographic distances (*in r = *0.29, *p* < .05; Figure S2b) as well as in significantly positive correlations between genetic and environmental distances (*r = *0.34*, p* < .01; Figure S2c).

### 3.4 Responses of genetic clusters toward climate change

**FIGURE 4:**
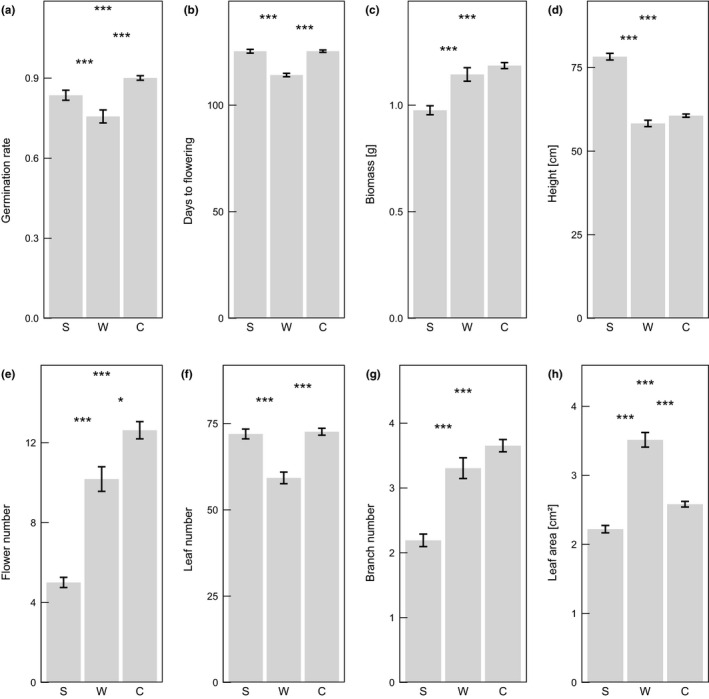
Phenotypic differences between genetic clusters (South (S), West (W), and central (C) European clusters as defined in Figure 3)

Analyses with mixed‐effect models revealed that genetic clusters—as defined by STRUCTURE—significantly differed in the number of germinating plants, in days to flowering, plant biomass, plant height, flower, leaf, and branch number, and in leaf area (Table 3; Figure 4). For germination rate and flower number, the average values for the plants in the Central cluster were significantly higher than in the other clusters (Figure 4a,e). Time to flowering was shortest in the West cluster, and plant biomass was lowest in the South cluster (Figure 4b,c). Plant height and leaf area showed a different pattern, with the tallest plants being found in the South cluster, and plants from the West cluster having the biggest leaves (Figure 4d,h). However, the number of leaves was lowest in the West cluster and plants from the South cluster possessed fewer branches (Figure 4f,g).

**FIGURE 5:**
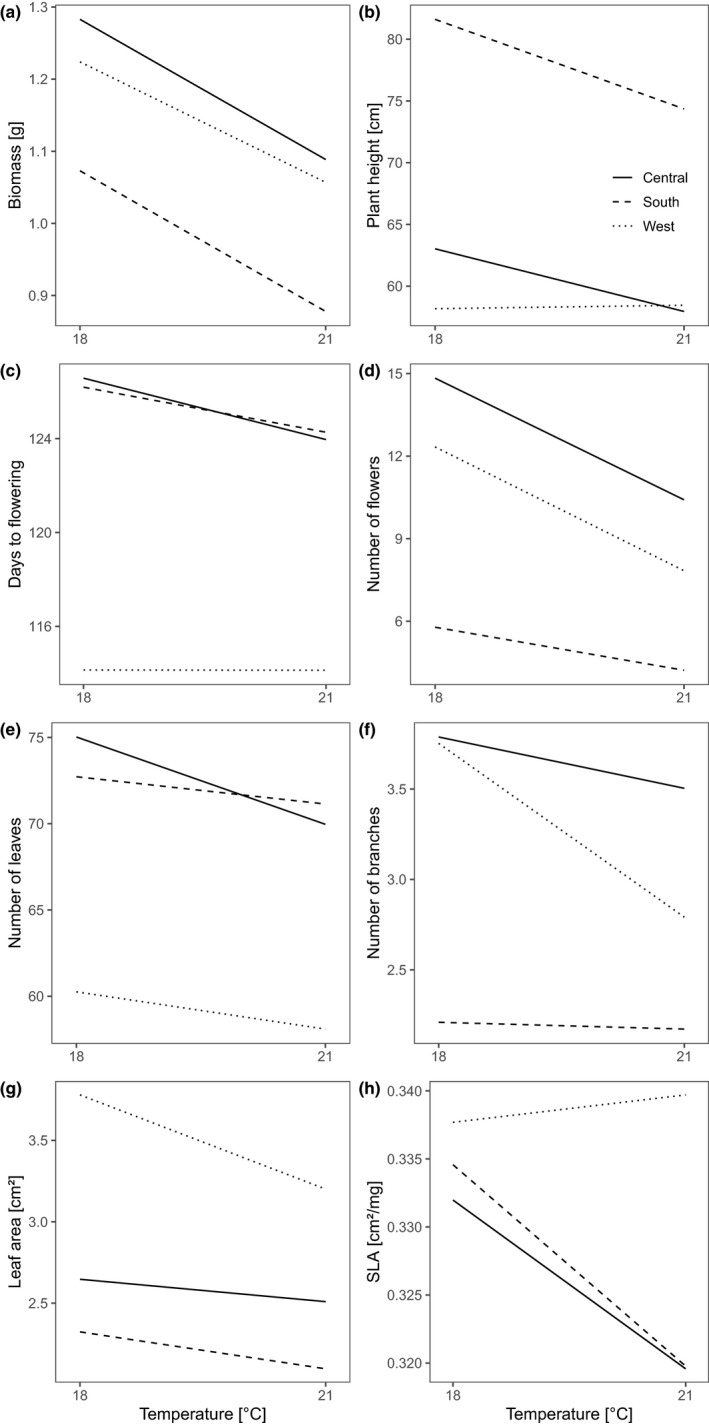
Reaction norms for temperature treatments of the three genetic clusters of *Silene vulgaris* (South, West, and central European clusters as defined in Figure 3). SLA, Specific leaf area

**FIGURE 6:**
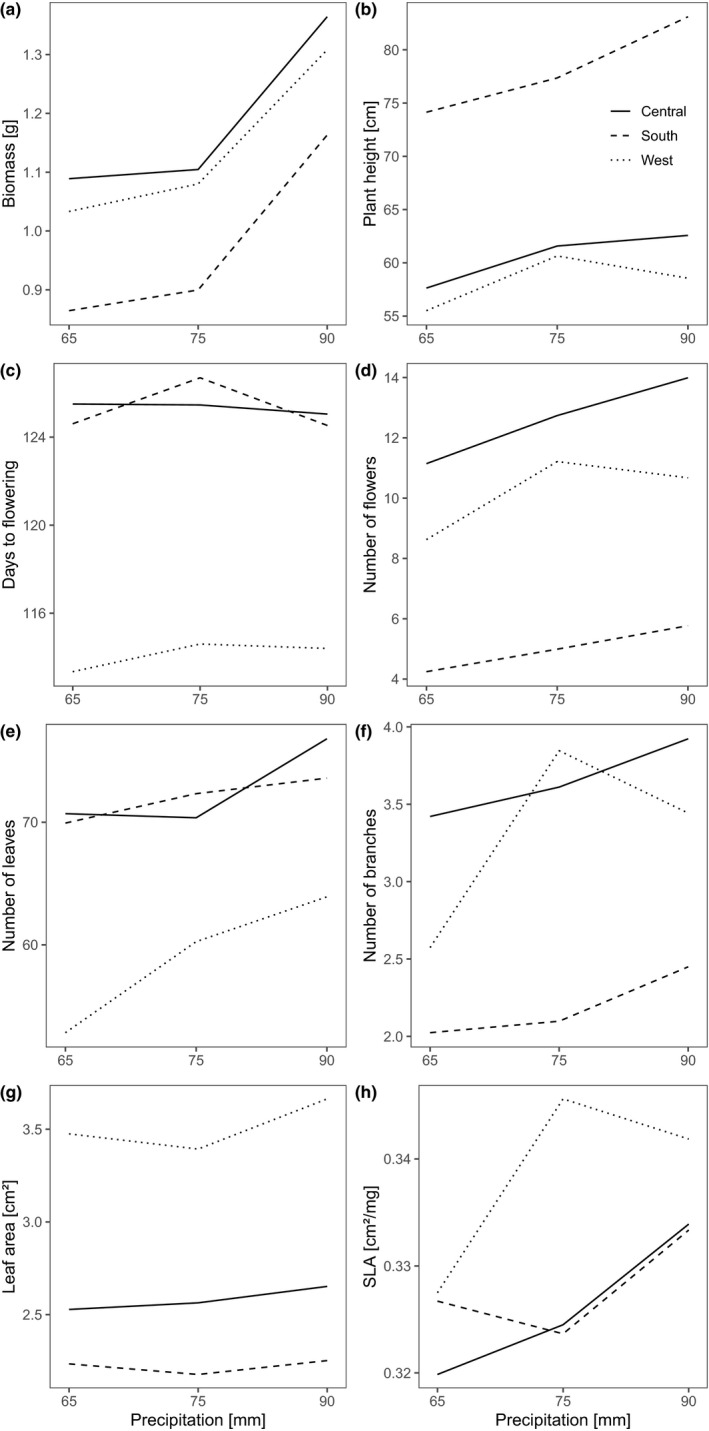
Reaction norms for precipitation treatments of the three genetic clusters of *Silene vulgaris* (South, West, and central European clusters as defined in Figure 3). SLA, Specific leaf area

There was no significant interaction between the genetic clusters and either temperature or precipitation conditions for six of the traits (germination, survival, flowering, biomass, days to flowering, and specific leaf area; Table 3). For the remaining five traits (leaf number, branch number, flower number, leaf area, and plant height), the three clusters differed in their responses to the temperature and/or precipitation treatments (Table 3). In the South and Central clusters, plant height strongly decreased with increasing temperature, whereas it slightly increased in the West cluster (Figure 5b). Number of leaves and branches and the leaf area decreased in all clusters with an increased temperature but showed a stronger response in the Central and West clusters, respectively (Figure 5e–g). The reaction norms of flower, leaf, and branch number differed in the three genetic clusters in response to the different precipitation treatments (Figure 6d–f): With increased precipitation, the number of flowers in the South and Central clusters was increased, whereas the West cluster showed a peak in flowers at 75 mm precipitation (Figure 6d). The number of leaves showed a decrease from 90 mm to 75 mm precipitation in all three clusters that further declined in the South and West clusters from 75 mm to 65 mm. The Central cluster showed a similar number of leaves under the 75 and 65 mm treatment (Figure 6e). For the number of branches, the genetic clusters revealed a similar reaction norm for the Central and South clusters, whereas the West cluster exhibited a strongly increased number of branches under the 75 mm treatment. For plant biomass, however, the three genetic clusters mainly showed a parallel pattern in reaction norms (Figures 5a and 6a).

## 4 DISCUSSION

How different plant species can adapt to a changing climate represents a crucial ecological and evolutionary question. As widely distributed species often possess many genetic lineages or subspecies (Van Rossum et al., 2018), revealing their population structure may help to predict their response to different environmental conditions at present and under future conditions (Collart et al., 2021). In many European plant species, a phylogeographic pattern can be found that resulted from the survival in different refugia during the last glacial maximum (LGM) (Bagnoli et al., 2016; Beatty & Provan, 2011; Krebs et al., 2019; Listl et al., 2017; Roces‐Díaz et al., 2018; Schwarzer & Joshi, 2019; Sebasky et al., 2016; Taberlet et al., 2012). During the LGM, Northern Europe was covered with glaciers that were also scattered in the mountainous regions of central Europe (Heyman et al., 2013). The climatic change associated with the LGM affected many plant species and drove their distribution to defined refugia forming biodiversity hotspots that also served as postglacial sources for recolonization (Hewitt, 2004; Morelli et al., 2016; Petit, 2003). The recent genetic lineages that were formed from these past events often show differential adaptation to environmental conditions (Prunier et al., 2012; Walden et al., 2020; Yan et al., 2019). Uncovering these population structures may thus facilitate the understanding of plant responses to a changing climate in the future.

In the present study, we used the model plant species *S. vulgaris* to uncover its genetic population structures within its European distributional range. Secondly, we analyzed whether the response to experimentally induced climate change differed between the genetic clusters detected.

### 4.1 The phylogeographic pattern and genetic diversity of *S. vulgaris*

The genome‐wide analysis of SNPs identified three major clusters originating from Southern, Western, and central Europe possibly representing a disruption pattern of the pan‐European species *S. vulgaris* during the last glacial maximum. Spanish (E1, E2, E3, E4) and French (F1, F2, F4, F5) *S. vulgaris* populations analyzed in this study appear in separate clusters. A similar pattern was also found in the population analysis by Sebasky et al. (2016) who also proposed a separate cluster for France in contrast to a cluster found on the Iberian Peninsula for *S. vulgaris*. Comparable population structures were also found for the closely related *Silene nutans* with a separate historic refugium on the Iberian Peninsula expanding toward south‐western France (Van Rossum et al., 2018). In the present phylogenetic analysis, the central European populations of *S. vulgaris* formed a third clade with comparably low resolution. Within this clade, no further geographic differentiation was possible with the data available. Interestingly, *S. vulgaris* populations from Sweden (S1, S2) did not stand out from the remaining central European populations. The two Swedish populations (S1 and S2) clustered together in a well‐supported branch within the third cluster. The phylogenetic analysis has shown that *S. vulgaris*, a species widely distributed in Europe, shows a considerable range of phylogenetic diversity but can be structured in three major clusters. This phylogenetic pattern can be of different origin. One possibility is that this pattern is a result of the recolonization of Europe from distinct refugia after the LGM. In the sampled *S. vulgaris* populations, indicators for genetic diversity (*H*_E_, *H*_O_, *π*) declined with increasing latitude. The most southern populations of *S. vulgaris* showed the highest values of observed heterozygosity. Similar observations were found for nucleotide diversity in the present study: Nucleotide diversity was lower in populations from higher latitudes compared to populations from lower latitudes. These findings may indicate that Southern European populations have been functioning as refugia during the LGM. During that time, populations in the south of Europe remained larger as they were not disrupted by snow‐covered areas or glaciers. When temperatures were increasing, again, a subsequent recolonization of central and northern Europe was likely to start from these southern refugia. The discovered pattern in *S. vulgaris* of decreased genetic diversity with increasing latitude is a commonly found pattern in different species after the last ice age (Beatty & Provan, 2011; Breen et al., 2009; Chung et al., 2018; Hewitt, 1999) and can be traced to the subdivision of populations in southern refugia and small population sizes during the recolonization. Overall, we detected three major genetic clusters in South, West, and central Europe for the sampled *S. vulgaris* populations whose genetic diversity decreased with increasing latitude. However, both, the genetic clusters and decreasing genetic diversity with latitude are only unspecific indicators of the populations’ evolutionary past. The recent genetic lineages may be the result of past disruptive population genetic events during the last ice age, and *S. vulgaris* may share the same glacial refugia with other *Silene* species (García‐Fernández et al., 2013; Meindl et al., 2016; Tausch, 2019; Taylor & Keller, 2007; Van Rossum et al., 2018). However, the hypotheses about *S. vulgaris*’ evolutionary past need further investigation, and for an unambiguous identification of the refugia during the LGM, further population genetic analyses are needed. Therefore, we suggest undertaking further distribution modeling to identify possible refugia and use coalescent‐based methods to reliably analyze the genetic variation presently found in *S. vulgaris* (Rosenberg & Nordborg, 2002; Sebasky et al., 2016).

Interestingly, we found for *S. vulgaris* that only observed heterozygosity significantly decreased with higher latitudes, whereas for *H*_E_ and *π,* we only found a decreasing trend. A possible explanation for this result is an increased inbreeding in northern populations. This could consequently lead to an increase in the number of homozygotes and thus a lower *H_O_*. However, at the same time, this extent of inbreeding did not lead to a loss of overall genetic diversity at the population level and could be the reason why He and π do not show this significant decrease. A similar case has been described by Bemmels and Dick (2018), where *H*_O_ was significantly decreased in southern populations of North American hickory tree species. In general, we found relatively low values of *F*_IS_ suggesting a strong outbreeding behavior of the populations. Although hermaphrodite individuals of S. vulgaris are self‐compatible, outcrossing is preferred as it leads to a higher fitness of offspring (Bailey & McCauley, 2006). Also, female individuals in *S. vulgaris* are clearly dependent on the pollination by hermaphrodites. This fact makes outbreeding a necessity at least for the female individuals of this species.

### 4.2 Environmental drivers of population structure

In our Mantel test analysis, the populations examined showed increased genetic differences with increasing geographic distance. This is a typical pattern for isolation by distance where stronger genetic differences are expected with an increasing distance between populations (Meirmans, 2012). More importantly, we found a significant positive correlation between geographic and environmental distance at the populations' sites of origin. As we included climatic factors in the analysis, we can conclude that those populations situated further apart from each other also experience stronger differences in climatic conditions (e.g., temperature or precipitation). These climatic conditions represent strong selective forces (Blackman et al., 2017; Moore et al., 2020; Santana et al., 2020). This is supported by the significant correlation between genetic and environmental distances in our study and underlines the likely importance of climatic factors as selection factors for *S. vulgaris*. In the present study, we were able to identify several fitness‐related traits (e.g., plant height, biomass, and number of flowers) and time to flowering that differed significantly among the three genetic lineages. Possibly, these trait differences are an adaptation to different environmental habitat conditions of the genetic lineages. It is known from *S*. *vulgaris* that it possesses several different ecotypes with strong genetic differentiation that are adapted to extremely unfavorable habitat conditions (i.e., heavy‐metal pollution) (Bratteler et al., 2006; Muszyńska et al., 2019). The exceptional adaptation of *S. vulgaris* to these extreme environments suggests that the species also shows adaptation to putatively less strong selective forces, that is, the different climate zones in continental Europe. In conclusion, we can state that populations of *S. vulgaris* show stronger between‐population genetic differences when their habitats are less similar.

### 4.3 Cluster differences in response to temperature and precipitation treatment

At the species level, *S. vulgaris* possesses a considerable phenotypic plasticity with regard to temperature and precipitation changes that likely helps this species to adapt (Kahl et al., 2019a). To investigate differences in the response toward a possible climate change scenario between the genetic clusters, we measured several fitness‐related traits that are known to respond to drought and temperature stress (Khan et al., 2015; Eziz et al., 2017; Meineri et al., 2020; Rucker et al., 1995; Wellstein et al., 2017; Zeiter et al., 2016).

The general response pattern on a population level has been described before (Kahl et al., 2019a). In the present analysis, we focused on putative differences in response to experimental climate change conditions among the genetic lineages. As an overall result, we found that the genetic clusters differed significantly in most of the plant traits examined. Furthermore, we showed that the populations’ environmental habitat conditions were correlated with the genetic diversity of the specimens we sampled. The differences in plant traits between the genetic clusters lead to the conclusion that *S. vulgaris* already shows adaptation toward different climatic conditions in Europe where the clusters are situated (South, West, and central Europe). With the currently ongoing climatic changes, mean temperatures are rising in Europe and precipitation patterns are changing drastically (Bindi & Olesen, 2011; Marx et al., 2018; Trnka et al., 2011). If we are interested in how species can adapt to these habitat changes, their genetic background has to be considered (Anderson et al., 2011; Bowles & Whelan, 1996; Corlett, 2017; McMahon et al., 2014; Vandergast et al., 2008). The inclusion of intraspecifc lineages is of strong importance here as it has been proven to substantially impact analyses on ecological niche modeling (Collart et al., 2021). Therefore, we included the phylogenetic data to investigate possible response differences in *S*. *vulgaris* to temperature increase and precipitation decrease from a possible climate change scenario. Our analysis revealed differences in the phenotypic response of the genetic clusters. The response of flower, leaf, and branch numbers as well as leaf area and plant height to the precipitation and temperature treatments differed significantly between the three genetic clusters. The illustration of phenotypic plasticity shows that in these traits one of the genetic clusters showed an opposing trait response (e.g., cluster West between 75 and 90 mm of precipitation). Because we used one climate change scenario for all *S. vulgaris* plants, it is possible that the highest precipitation treatment is out of the optimum range for the West cluster. Hence, the number of flowers or branches decreased with an increased precipitation of 90 mm. With the knowledge we gained from the phylogeographic analysis, it would be of interest to expose plants from the different clusters to differing climate change scenarios specified for the region they originate from. This approach could further facilitate the understanding of climate adaption in the genetic lineages of *S. vulgaris*. In general, we found that under most treatments, the clusters’ responses point in the same direction (see reaction norms; Figures 5 and 6). Thus, the genetic clusters we found in European *S. vulgaris* populations mostly did not show contradicting differences when responding to changing temperature and precipitation conditions. Climate change may thus not favor one or the other phylogenetic cluster as all three seem to have a comparable basis of adaptability. Hence, even though we found potential differences in the genetic diversity and habitat adaptations at a population level, overall *S. vulgaris* does not show striking differences in its response to an experimental climate change scenario. An earlier study suggests that *S. vulgaris* possesses a considerable amount of phenotypic plasticity toward temperature and precipitation regimes (Kahl et al., 2019a). Hence, in the case of this widely distributed species, phenotypic plasticity may play an important role in adaptation processes. In other species, it has also been shown that phenotypic plasticity provides a strong mechanism to mitigate negative effects of climate change (Frank et al., 2017; Kingsolver & Buckley, 2017; Peterson et al., 2018; Richardson et al., 2017). In this context, knowledge on genetic markers of phenotypic plasticity for *S. vulgaris* would help to verify this hypothesis.

In the future, climate change will either lead to a shift in distribution patterns in plants or will force them to adapt locally (Ahrens et al., 2020; Anderson & Wadgymar, 2020; He et al., 2019; Metz et al., 2020). The present study used a comprehensive approach including a pan‐European sampling and a greenhouse experiment on climate change adaptation paired with a population genetic analysis to understand the interaction of population genetics and current trait responses. The results revealed three genetic clusters for *S. vulgaris* showing distinct trait differences. However, the three clusters did not show major differences in their response to experimental climate change conditions. Hence, for the widely distributed *S. vulgaris,* phenotypic plasticity seems to represent an important aspect when facing the obstacles of rapid climate change.

## CONFLICT OF INTEREST

The authors declare that they have no conflict of interest.

## AUTHOR CONTRIBUTIONS

**Sandra M. Kahl:** Conceptualization (lead); Data curation (lead); Formal analysis (lead); Investigation (lead); Methodology (lead); Visualization (lead); Writing‐original draft (lead); Writing‐review & editing (lead). **Christian Kappel:** Formal analysis (supporting); Visualization (supporting); Writing‐original draft (supporting); Writing‐review & editing (supporting). **Jasmin Joshi:** Conceptualization (supporting); Methodology (supporting); Supervision (lead); Writing‐original draft (supporting); Writing‐review & editing (supporting). **Michael Lenhard:** Conceptualization (supporting); Methodology (supporting); Supervision (lead); Writing‐original draft (supporting); Writing‐review & editing (supporting).

## Supporting information

Figure S1

Figure S2

Tables S1‐S2

## Data Availability

Plant trait data from greenhouse experiment is available from the Dryad Digital Repository: https://doi.org/10.5061/dryad.0p2ngf227. ddRAD sequencing data are available at NCBI SRA under accession number PRJNA591058 (from December 31, 2021, onwards). Data can be reviewed under https://dataview.ncbi.nlm.nih.gov/object/PRJNA591058?reviewer=chgqlnh9gel7leca3pch0ula0p.
