# Supplementary figures and images for: Phylogeography of a widely distributed plant species reveals cryptic genetic lineages with parallel phenotypic responses to warming and drought conditions
Source: Ecol Evol. 2021 Sep 9;11(20):13986–4002. doi: 10.1002/ece3.8103 (PMC8525116; doi:10.1002/ece3.8103)

Color Key

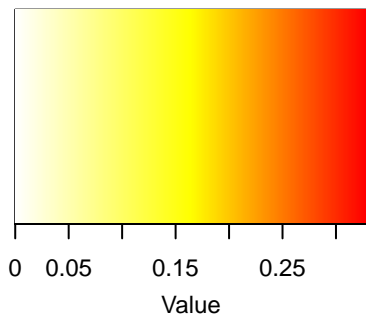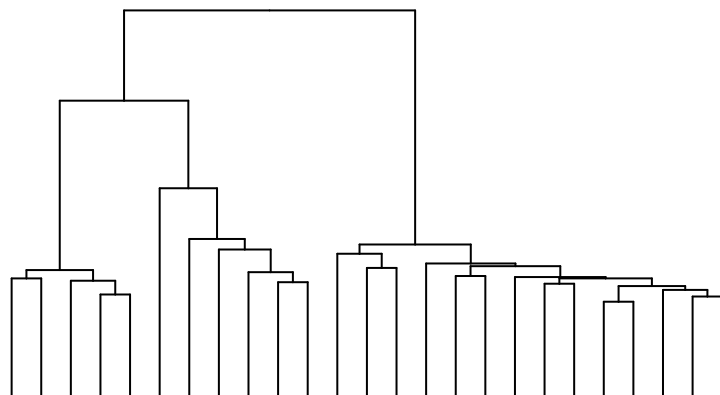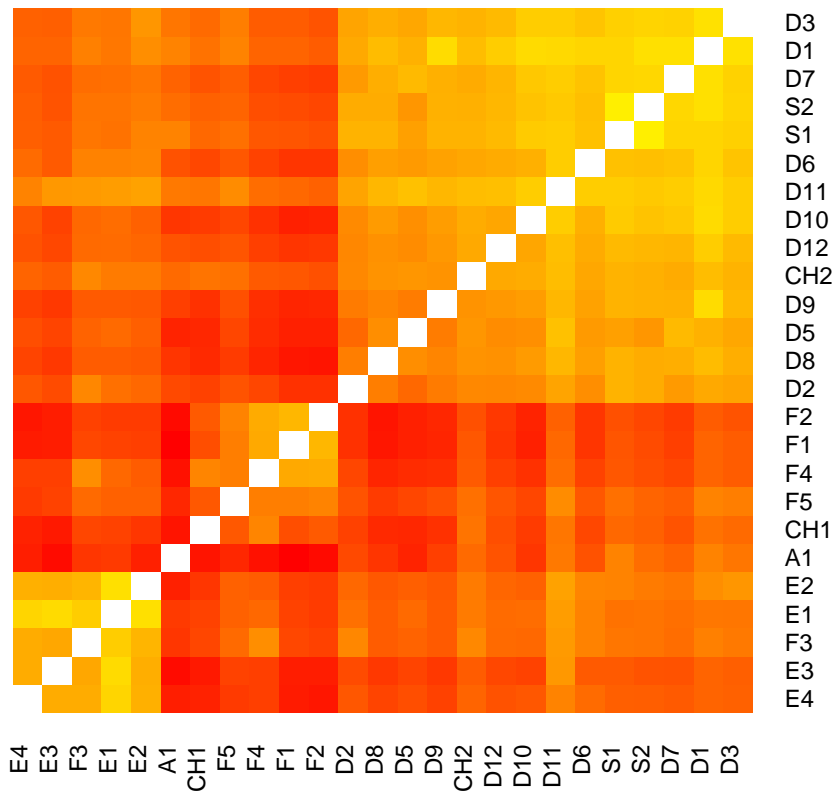

Supplement: Supplementary file 1 — Figure S1 [file ECE3-11-13986-s001.pdf]

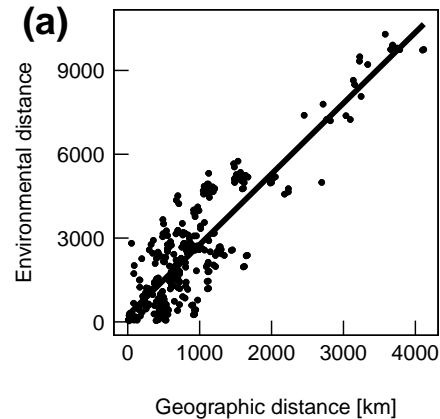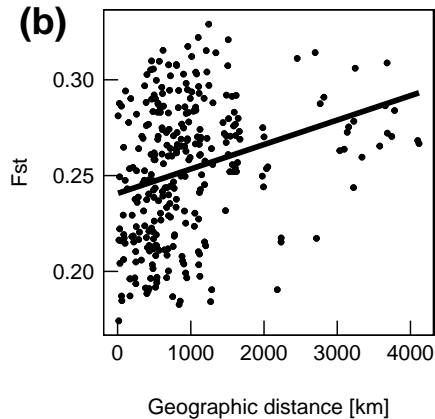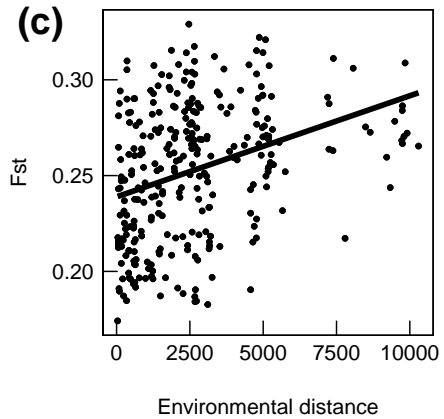

Supplement: Supplementary file 2 — Figure S2 [file ECE3-11-13986-s002.pdf]
