# Supplementary material for: Phylogeography of a widely distributed plant species reveals cryptic genetic lineages with parallel phenotypic responses to warming and drought conditions
Source: Ecol Evol. 2021 Sep 9;11(20):13986–4002. doi: 10.1002/ece3.8103 (PMC8525116; doi:10.1002/ece3.8103)
Supplement: Supplementary file 3 — Tables S1‐S2 [file ECE3-11-13986-s003.docx]

**Table S1.** The 48 EcoRI barcode adapters with unique 5 base pair sequences and the MspI and Illumina PCR oligo sequences used in this study.

**Table S2.** Climate data from the sampling sites of the *Silene vulgaris* populations obtained from WorldCim (Fick and Hijmans, 2017). bio_1: annual mean temperature, bio_2: mean diurnal range (mean of monthly (max temp - min temp)), bio_3: isothermality (bio_2/bio_7), bio_4: temperature seasonality (standard deviation), bio_5: maximum temperature warmest month, bio_6: minimum temperature coldest month, bio_7: temperature annual range (bio_5-bio_6), bio_8: mean temperature of wettest quarter, bio_9: mean temperature of driest quarter, bio_10: mean temperature of warmest quarter, bio_11: mean temperature of coldest quarter. All temperature variables are in °C.

**Table S2 (continued).** Climate data from the sampling sites of the *Silene vulgaris* populations obtained from WorldCim (Fick and Hijmans, 2017). bio_12: annual mean precipitation, bio_13: precipitation wettest month, bio_14: precipitation driest month, bio_15: precipitation seasonality (coefficient of variation), bio_16: precipitation wettest quarter, bio_17: precipitation driest quarter, bio_18: precipitation warmest quarter, bio_19: precipitation coldest quarter, s_rad: solar radiation in kJ/(m²*d), w_sp: wind speed in m*s^-1^, w_v_pr: water vapor pressure in kPa. All precipitation variables are in mm.

**Figure S1**. F_ST_ population dendrogram and heat map based on F_ST_ values among the 25 Silene vulgaris populations. The heat map color illustrates the F_ST_ values.

**Figure S2**. Relationship between (a) environmental (Euclidean) and geographic distances (r=0.89, *p<0.001*), (b) genetic (F_ST_) and geographic distances (r=0.29, *p*<0.05), and (c) genetic (F_ST_) and environmental (Euclidean) distances (r=0.34, *p*<0.01) of the 25 Silene vulgaris populations.

**Table S1**

| Name | Sequence |
| --- | --- |
| EcoRI |  |
| EcoRI_P1.1.1 | GCATG |
| EcoRI_P1.1.2 | AACCA |
| EcoRI_P1.1.3 | CGATC |
| EcoRI_P1.1.4 | TCGAT |
| EcoRI_P1.1.5 | TGCAT |
| EcoRI_P1.1.6 | CAACC |
| EcoRI_P1.1.7 | GGTTG |
| EcoRI_P1.1.8 | AAGGA |
| EcoRI_P1.1.9 | AGCTA |
| EcoRI_P1.1.10 | ACACA |
| EcoRI_P1.1.11 | AATTA |
| EcoRI_P1.1.12 | ACGGT |
| EcoRI_P1.1.13 | ACTGG |
| EcoRI_P1.1.14 | ACTTC |
| EcoRI_P1.1.15 | ATACG |
| EcoRI_P1.1.16 | ATGAG |
| EcoRI_P1.1.17 | ATTAC |
| EcoRI_P1.1.18 | CATAT |
| EcoRI_P1.1.19 | CGAAT |
| EcoRI_P1.1.20 | CGGCT |
| EcoRI_P1.1.21 | CGGTA |
| EcoRI_P1.1.22 | CGTAC |
| EcoRI_P1.1.23 | CGTCG |
| EcoRI_P1.1.24 | CTGAT |
| EcoRI_P1.1.25 | CTGCG |
| EcoRI_P1.1.26 | CTGTC |
| EcoRI_P1.1.27 | CTTGG |
| EcoRI_P1.1.28 | GACAC |
| EcoRI_P1.1.29 | GAGAT |
| EcoRI_P1.1.30 | GAGTC |
| EcoRI_P1.1.31 | GCCGT |
| EcoRI_P1.1.32 | GCTGA |
| EcoRI_P1.1.33 | GGATA |
| EcoRI_P1.1.34 | GGCCA |
| EcoRI_P1.1.35 | GGCTC |
| EcoRI_P1.1.36 | GTAGT |
| EcoRI_P1.1.37 | GTCCG |
| EcoRI_P1.1.38 | GTCGA |

**Table S1 (continued)**

| Name | Sequence |
| --- | --- |
| EcoRI_P1.1.39 | TACCG |
| EcoRI_P1.1.40 | TACGT |
| EcoRI_P1.1.41 | TAGTA |
| EcoRI_P1.1.42 | TATAC |
| EcoRI_P1.1.43 | TCACG |
| EcoRI_P1.1.44 | TCAGT |
| EcoRI_P1.1.45 | TCCGG |
| EcoRI_P1.1.46 | TCTGC |
| EcoRI_P1.1.47 | TGGAA |
| EcoRI_P1.1.48 | TTACC |
|  |  |
| MspI |  |
| MspI_P2.1 | GTGACTGGAGTTCAGACGTGTGCTCTTCCGATCT |
| MspI_P2.2 | /5Phos/CGAGATCGGAAGAGCGAGAACAA |
|  |  |
| Illumina |  |
| PCR1 | AATGATACGGCGACCACCGAGATCTACACTCTTTCCCTACACGACG |
| PCR2_Idx_1_ATCACG | CAAGCAGAAGACGGCATACGAGATCGTGATGTGACTGGAGTTCAGACGTGTGC |
| PCR2_Idx_2_CGATGT | CAAGCAGAAGACGGCATACGAGATACATCGGTGACTGGAGTTCAGACGTGTGC |
| PCR2_Idx_3_TTAGGC | CAAGCAGAAGACGGCATACGAGATGCCTAAGTGACTGGAGTTCAGACGTGTGC |
| PCR2_Idx_4_TGACCA | CAAGCAGAAGACGGCATACGAGATTGGTCAGTGACTGGAGTTCAGACGTGTGC |
| PCR2_Idx_5_ACAGTG | CAAGCAGAAGACGGCATACGAGATCACTGTGTGACTGGAGTTCAGACGTGTGC |
| PCR2_Idx_6_GCCAAT | CAAGCAGAAGACGGCATACGAGATATTGGCGTGACTGGAGTTCAGACGTGTGC |
| PCR2_Idx_7_CAGATC | CAAGCAGAAGACGGCATACGAGATGATCTGGTGACTGGAGTTCAGACGTGTGC |
| PCR2_Idx_8_ACTTGA | CAAGCAGAAGACGGCATACGAGATTCAAGTGTGACTGGAGTTCAGACGTGTGC |

**Table S2**

| Population | bio_1 | bio_2 | bio_3 | bio_4 | bio_5 | bio_6 | bio_7 | bio_8 | bio_9 | bio_10 | bio_11 |
| --- | --- | --- | --- | --- | --- | --- | --- | --- | --- | --- | --- |
| A1 | 5.6 | 8.6 | 31.7 | 692.9 | 18.2 | -8.9 | 27.1 | 14.0 | -3.0 | 14.0 | -3.0 |
| CH1 | 1.0 | 7.1 | 32.9 | 547.7 | 12.2 | -9.2 | 21.4 | 8.1 | -4.8 | 8.1 | -4.8 |
| CH2 | 11.2 | 9.4 | 32.1 | 728.2 | 25.4 | -3.9 | 29.3 | 15.0 | 2.3 | 20.3 | 2.3 |
| D1 | 9.9 | 7.1 | 27.6 | 696.8 | 22.0 | -3.8 | 25.8 | 18.6 | 5.2 | 18.6 | 1.5 |
| D2 | 8.8 | 7.3 | 30.4 | 613.3 | 20.2 | -3.9 | 24.1 | 16.6 | 4.4 | 16.6 | 1.5 |
| D3 | 9.7 | 7.4 | 28.1 | 697.0 | 22.1 | -4.3 | 26.4 | 18.4 | 5.1 | 18.4 | 1.3 |
| D5 | 10.2 | 7.8 | 30.3 | 649.3 | 22.3 | -3.6 | 25.9 | 16.8 | 3.5 | 18.3 | 2.4 |
| D6 | 8.6 | 8.3 | 30.4 | 687.5 | 21.4 | -5.8 | 27.2 | 15.7 | 4.1 | 17.1 | 0.3 |
| D7 | 8.5 | 7.9 | 29.1 | 683.5 | 21.1 | -5.9 | 27.0 | 17.0 | 3.8 | 17.0 | 0.2 |
| D8 | 8.7 | 7.5 | 28.0 | 696.2 | 21.2 | -5.7 | 26.9 | 17.4 | 1.2 | 17.4 | 0.3 |
| D9 | 9.2 | 7.6 | 28.5 | 687.7 | 21.6 | -4.9 | 26.5 | 17.8 | 4.6 | 17.8 | 0.9 |
| D10 | 9.6 | 8.2 | 30.2 | 701.3 | 22.4 | -4.9 | 27.3 | 18.3 | 2.4 | 18.3 | 1.0 |
| D11 | 10.5 | 8.2 | 30.9 | 668.7 | 22.9 | -3.6 | 26.5 | 17.3 | 3.7 | 18.9 | 2.5 |
| D12 | 9.5 | 7.3 | 28.8 | 677.2 | 21.4 | -4.1 | 25.5 | 17.9 | 2.5 | 17.9 | 1.3 |
| E1 | 15.0 | 12.3 | 39.4 | 684.6 | 30.1 | -1.2 | 31.3 | 15.5 | 23.7 | 23.7 | 6.9 |
| E2 | 12.1 | 11.3 | 37.3 | 667.5 | 27.2 | -3.2 | 30.4 | 13.4 | 5.4 | 21.0 | 4.6 |
| E3 | 14.1 | 12.0 | 38.9 | 678.9 | 28.9 | -2.0 | 30.9 | 14.7 | 7.4 | 22.8 | 6.3 |
| E4 | 14.5 | 6.2 | 41.3 | 323.7 | 22.5 | 7.5 | 15.0 | 11.2 | 18.3 | 18.9 | 11.0 |
| F1 | 12.1 | 8.8 | 38.9 | 519.6 | 22.8 | 0.2 | 22.6 | 6.7 | 18.6 | 18.6 | 5.9 |
| F2 | 12.4 | 11.4 | 38.9 | 661.2 | 27.1 | -2.3 | 29.4 | 13.0 | 20.9 | 20.9 | 4.7 |
| F3 | 11.6 | 9.0 | 36.8 | 572.7 | 23.2 | -1.1 | 24.3 | 8.2 | 18.8 | 18.8 | 4.8 |
| F4 | 11.5 | 9.3 | 33.7 | 665.6 | 24.7 | -2.9 | 27.6 | 11.7 | 3.5 | 19.9 | 3.5 |
| F5 | 11.7 | 8.4 | 31.9 | 649.7 | 24.3 | -2.0 | 26.3 | 11.9 | 4.0 | 20.0 | 4.0 |
| S1 | 7.2 | 7.5 | 30.3 | 655.2 | 19.3 | -5.5 | 24.8 | 15.1 | 1.8 | 15.7 | -0.2 |
| S2 | 7.3 | 7.8 | 29.9 | 678.0 | 20.1 | -6.1 | 26.2 | 15.2 | 1.7 | 16.1 | -0.4 |

**Table S2 (continued)**

| Pop-ulation | bio_12 | bio_13 | bio_14 | bio_15 | bio_16 | bio_17 | bio_18 | bio_19 | s_rad | w_sp | w_v_pr |
| --- | --- | --- | --- | --- | --- | --- | --- | --- | --- | --- | --- |
| A1 | 977 | 140 | 37 | 45 | 402 | 124 | 402 | 124 | 11861 | 1.9 | 0.7 |
| CH1 | 1965 | 211 | 117 | 17 | 607 | 390 | 607 | 398 | 12866 | 4.1 | 0.5 |
| CH2 | 1473 | 171 | 61 | 32 | 476 | 215 | 460 | 215 | 12618 | 1.4 | 1.1 |
| D1 | 575 | 65 | 36 | 19 | 176 | 117 | 176 | 137 | 9993 | 3.7 | 0.9 |
| D2 | 792 | 81 | 45 | 17 | 223 | 159 | 223 | 193 | 9607 | 3.6 | 0.9 |
| D3 | 577 | 69 | 35 | 20 | 182 | 116 | 182 | 134 | 10061 | 3.7 | 0.9 |
| D5 | 574 | 64 | 36 | 18 | 181 | 116 | 167 | 125 | 10411 | 2.8 | 1.0 |
| D6 | 743 | 83 | 48 | 18 | 234 | 154 | 227 | 165 | 10695 | 3.0 | 0.9 |
| D7 | 538 | 70 | 29 | 28 | 189 | 95 | 189 | 106 | 10098 | 3.2 | 0.9 |
| D8 | 510 | 62 | 27 | 26 | 176 | 94 | 176 | 101 | 10158 | 2.9 | 0.9 |
| D9 | 558 | 65 | 36 | 19 | 172 | 115 | 172 | 133 | 10151 | 4.0 | 0.9 |
| D10 | 879 | 110 | 47 | 27 | 304 | 151 | 304 | 163 | 11268 | 2.1 | 1.0 |
| D11 | 601 | 71 | 36 | 21 | 198 | 116 | 182 | 122 | 10569 | 2.7 | 1.0 |
| D12 | 1094 | 132 | 57 | 29 | 386 | 183 | 386 | 191 | 11445 | 2.0 | 1.0 |
| E1 | 366 | 49 | 10 | 37 | 121 | 62 | 62 | 72 | 15336 | 2.6 | 1.2 |
| E2 | 662 | 77 | 33 | 24 | 200 | 126 | 151 | 134 | 15263 | 3.1 | 0.9 |
| E3 | 490 | 58 | 21 | 28 | 153 | 93 | 106 | 94 | 15140 | 2.5 | 1.1 |
| E4 | 432 | 80 | 1 | 78 | 216 | 6 | 21 | 186 | 19890 | 6.2 | 1.0 |
| F1 | 773 | 88 | 39 | 28 | 259 | 121 | 121 | 243 | 12508 | 3.9 | 1.1 |
| F2 | 739 | 94 | 32 | 24 | 235 | 142 | 142 | 173 | 14923 | 3.3 | 1.0 |
| F3 | 770 | 76 | 51 | 13 | 215 | 159 | 159 | 201 | 12112 | 3.4 | 1.1 |
| F4 | 971 | 108 | 65 | 19 | 299 | 200 | 222 | 200 | 12677 | 2.9 | 1.0 |
| F5 | 838 | 95 | 50 | 21 | 259 | 159 | 205 | 159 | 12715 | 3.1 | 1.0 |
| S1 | 524 | 56 | 32 | 19 | 156 | 101 | 147 | 123 | 10149 | 4.8 | 0.9 |
| S2 | 509 | 55 | 29 | 20 | 157 | 96 | 146 | 117 | 10171 | 4.7 | 0.9 |
